# Perspectives of Family Caregivers Confronted With Care-Resistant Behavior From Persons With Dementia

**DOI:** 10.1093/geroni/igaa057.813

**Published:** 2020-12-16

**Authors:** Rita Jablonski, Vicki Winstead, David Geldmacher

**Affiliations:** 1 UAB School of Nursing, Birmingham, Alabama, United States; 2 University of Alabama at Birmingham, Alabaster, Alabama, United States; 3 UAB School of Medicine, Birmingham, Alabama, United States

## Abstract

Problem: Care-resistant behavior is often bundled with other behavioral symptoms of dementia, but it is a unique behavior requiring targeted interventions. Purpose: To describe the experiences of caregivers receiving online coaching to manage care-resistant behaviors exhibited by persons with dementia. Design: Qualitative. Sample & Procedure: 20 caregivers (12 female, 8 male) were recruited from Memory Disorders and Geriatrics clinics to participate in 6 weeks of online coaching sessions delivered by a doctorally prepared nurse practitioner. Coaching sessions were recorded and transcribed. NVivo12 software was used to manage the thematic analyses. Results: Caregivers followed a general trajectory. They initially reported feelings of anger, frustration, and guilt. They believed that the person with dementia was purposefully “being stubborn and mean.” As the coaching sessions progressed, these negative emotions and the attributions of intent altered. By the conclusion of the six weeks, caregivers expressed feelings of success and ingenuity in applying coaching strategies. Conclusion: Online coaching is an effective way to individualize strategies that enable the caregiver to manage and reduce care-resistant behavior. Implications: The use of a doctorally-prepared nurse practitioner to deliver coaching, while effective, is not sustainable. Next steps include developing a coaching training program that could be embedded into existing community resources for community-dwelling caregivers. Limitations: Participants were limited to referrals from two clinics in the same institution.

